# Measuring Sensors Calibration in Worm Gear Rolling Testers

**DOI:** 10.3390/s20113148

**Published:** 2020-06-02

**Authors:** Marcos Pueo, Raquel Acero, Ángel Gracia, Jorge Santolaria

**Affiliations:** 1 Centro Universitario de la Defensa, Academia General Militar, Ctra. Huesca s/n, 50090 Zaragoza, Spain; agraciar@unizar.es; 2 Department of Design and Manufacturing Engineering, University of Zaragoza, María de Luna 3, 50018 Zaragoza, Spain; racero@unizar.es (R.A.); jsmazo@unizar.es (J.S.)

**Keywords:** calibration, numerical compensation, gear metrology, meshing point, worm gear

## Abstract

The ISO standard regulating gear-rolling measurement does not specify in detail the calibration and verification procedures for this type of equipment. This may be one of the reasons for the lack of reproducibility in these rolling tests. The uncertainty budget method, which is the most appropriate way to know the accuracy of this dynamic measurement, shows that the measuring sensors’ accuracy is only a part of the total measurement process uncertainty. In this work, a new calibration and verification procedure for a worm gear rolling tester is presented, based on machine tool, coordinate measuring machine and gear measuring instruments’ calibration techniques. After compensating numerically for the measuring instruments, it has been evaluated how the error components of each movement affect the meshing point, a fundamental factor to ensure a good gear transmission. The study shows that there are unintentional position variations, not detected by the measuring sensors, that have to be identified and quantified in the calibration for their later inclusion in the uncertainty budget. In this way, the measurement uncertainty could be reduced, and thus improve the reproducibility of these testers, as a preliminary stage to the development of optimized rolling measurement equipment to solve current limitations.

## 1. Introduction

Rolling tests are functional tests where the quality grade of a gear unit is determined by rolling it against a higher quality (precision) master gear. They are also used to check complete transmissions and even to match gears in their optimal position [1,2,3,4,5]. These tests are presented as the fastest and most complete way to check the behaviour of a gear or a transmission since it is not limited to checking the geometry of a few randomly selected teeth but of the complete set. From its results, both geometric errors (profile, pitch, cumulative pitch and eccentricity errors) and manufacturing problems (misalignments in the axes, poor clamping, handling shocks, etc.) can be identified. In addition, they allow for the verification of parallel, bevel and worm gears. For this reason, they have been widely used in industry for decades, mainly as controls during the production process [6]. Nowadays, there is a growing interest in studies and developments of new gear measurement techniques and instruments, based on rolling principles, due to its potential in industry [7,8,9].

**Figure 1:**
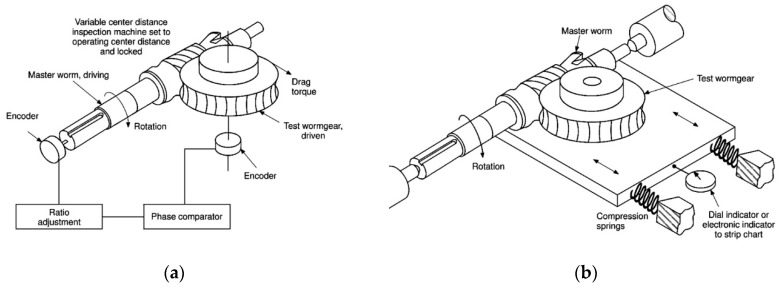
Worm gear rolling tests working principle: (**a**) single flank rolling test; (**b**) double flank rolling test. (Reprinted from [10] with permission of the copyright holder, AGMA).

Gear rolling tests are also referred to as composite tests since their results do not come from an individual unit measurement but from the combination of thousands of continuous measurements depending on the type of test. In fact, the rolling parameters are obtained from a sinusoidal-type graph that is decomposed, usually by means of the Fourier transform, to extract the individual information of each tooth. The gear quality is assigned according to the worst quality of any of its rolling parameters. In the single-flank rolling test (tangential composite), the transmission error is checked at the nominal position of a gear by comparing the theoretical angle of rotation with the angle turned by the test gear. In the double-flank rolling test (radial composite), the change in the centre distance between the gears is checked when they are run in without play at a distance less than the nominal distance. The two types of rolling tests are based on different measuring principles and, therefore, so are the interpretations of their results according to VDI/VDE 2608 [1]. This type of gear measurement is suitable for cylindrical, bevel and worm gears. In particular, the gear arrangement and operating principles for single- and double-flank worm gears are shown in Figure 1a,b, respectively, where the master worm drags the test worm gear.

### 1.1. Calibration and Traceability

In the 1990s, the National Gear Metrology Laboratory (NGML) in Great Britain found poor measurement techniques, insufficient calibration routines and lack of traceability in gear manufacturing companies. Therefore, the British Gear Association (BGA) published a series of codes of practice (DUCOP) that established the basis for calibration standards in gear measurement [11]. A similar study was later carried out in the United States, resulting in the publication of several standards by American Gear Manufacturers Association (AGMA) that included information about calibration conditions, accuracy requirements and determination of uncertainty in gear measurement [12,13,14,15]. In 2002, AGMA 931-A2 “Calibration of Gear Measuring Instruments and Their Application to the Inspection of Product Gears” [16] brought together all the information from its predecessors together with the input of the BGA codes of practice. All this work has been the basis for the development of the current international standards for evaluation and instrumentation of gear measurement ISO 18653:2003 [17] and ISO/TR 10064-5:2005 [18] by the ISO TC 60/WG2 working group. In general, these standards recommend a full initial calibration and subsequent regular follow-up inspections to ensure proper gear axes alignment and accuracy of the measurement system. In addition, they also recommend the use of traceable calibrated devices that can verify the complete working volume of the machine.

After this previous experience, the AGMA Calibration Committee decided that a similar standardization was also necessary for the evaluation methods of the double-flank testing equipment. Therefore, ANSI/AGMA 2116-A05 “Evaluation of Double Flank Testers for Radial Composite Measurement of Gears” [19] and AGMA 935-A05 “Recommendations Relative to the Evaluation of Radial Composite Gear Double Flank Testers” [20] were published in 2005. These standards provide general guidelines for the evaluation and qualification of these devices, as well as some methods for the estimation of their measurement uncertainty. However, at present, not only is there no equivalent international standard yet, but there is not even any other similar reference standard that collects information about the calibration and verification of this type of single or double-flank gear rolling test equipment.

On the other hand, the term “traceability” implies an unbroken chain of comparisons from the measurements taken in the workshop to the primary devices of the national reference laboratories with all the established uncertainties [17,19]. Currently, there are only three certified primary laboratories in the world with the capability to provide traceability of gear devices that meet the specifications of ISO/IEC 17025 [21]: the National Institute of Standards and Technology (NIST) in the United States, the National Institute of Advanced Industrial Science and Technology (AIST) in Japan and the Nationale Physikalisch Technische Budesanstalt (PTB) in Germany [22]. In 2004, the data of the first international comparison of measurements of geometrical parameters of involute gears, organized and coordinated by the UK NGML, were published. The results were accepted as a key comparison and served as a mutual acceptance for the measurands among the national metrological institutes of Germany, USA and UK [23]. During the last few years, the PTB, at the proposal of the European Association of National Metrology Institutes (EURAMET), has led to two new interlaboratory comparisons to try to guarantee the compatibility of profile, helix and pitch measurements at international level [24,25]. Even so, these laboratories can only give limited traceability according to the standard artifacts they have and the usual parameters of gear measurement (profile, helix and pitch), quantifying the measurement uncertainty according to ISO 18653:2003 [17].

However, as far as the rolling parameters are concerned, none of them is accredited as there are no national reference rolling standards that provide a unique traceability procedure. Therefore, no interlaboratory comparisons of rolling tests have been made, even though evidence shows that identical results are not obtained when checking the same gear on different equipment even when the performance conditions are maintained [26,27,28]. However, there is repeatability in the rolling equipment, which makes the process traceable [29].

### 1.2. Rolling Test Measurement Uncertainty

Rolling tests are dynamic measurements that are subject to a large number of sources of error that make measurement evaluation difficult. The main reasons are: (i) the measurement is not a single dimension although the result is, by meshing two gears together; (ii) a large number of readings are handled which subsequently have to be filtered and processed; (iii) micrometric values are evaluated, which are dimensional ranges where any remaining material, dirt or surface imperfections may distort the inspection; and (iv) the reference artifacts are limited by the dimensions, geometries and dispositions of the different types of gears to be verified. All this, together with the lack of clear guidelines in the calibration of the equipment, explains the low reproducibility that still exists today in this type of tests.

In this situation, it is difficult to know the accuracy of the measurement process even knowing the accuracy of the measuring instruments. Uncertainty budget analysis is the best way to validate this dynamic measurement system [30] where sources of error are quantified, compensated where possible and their effects considered in the estimation of uncertainty [31].

Annex A of ANSI/AGMA 2116-A05 [19], extracted from ISO 18653:2003 [17], proposes several expressions to estimate the uncertainty of measurement in double-flank rolling equipment according to the methods of decomposition, substitution and comparison. However, it does not show any expression for applying the uncertainty budget method according to Guide to the Expression of Uncertainty in Measurement (GUM) guidelines [32]. Therefore, two expressions have recently been developed following this method for the estimation of uncertainty of a single-flank [33] and double-flank worm gear rolling machine [34]. This method could be further extended to the development of new gear rolling test equipment after the identification of critical design points.

They divide the uncertainty contributions into the different actions that are performed during a gear-rolling test: initial calibration, displacement to the nominal test position, assembly of the gears and execution of the test. Each of them is later broken down in terms of individual errors and repeatability, which can be quantified. The input data for calculating the measurement uncertainty of the gear-rolling tester must be experimentally obtained from the calibration and characterization of the tester.

In this work, we present the calibration and evaluation procedure of a single and double-flank rolling worm gear tester, analysing in detail the influence of the measurement sensors used on the accuracy of the equipment. The study shows the limitations of the current measurement systems, proposing a tool that allows the measurement uncertainty to be reduced, as a previous step to the development of a new type of rolling measurement equipment. In the absence of clear standards, the calibration procedure has been based on techniques commonly used for the calibration of machine tools (MTs), coordinate measuring machines (CMMs) and gear measurement instruments (GMIs) [31,35,36,37,38] in addition to following, as far as possible, the recommendations described by the aforementioned standards for the evaluation of gear measurement equipment [17,18,19,20]. In this way, it is possible to determine both the residual values of the numerical compensation of the measurement sensors and the errors in the meshing point affecting the rolling parameters and that the sensors could not detect.

## 2. Calibration and Evaluation of a Worm Gear Rolling Tester

Any calibration procedure for GMIs should be performed according to the recommendations established by the code of inspection practice ISO/TR 10064-5:2005 [18]. Some of these recommendations are also included in the AGMA 935-A05 [20] standard for the assessment of double-flank rolling test equipment, and are partially also applicable to single-flank, as both standards are based on AGMA 931-A2 [16]. They stress that it is important that the calibration process is carried out under normal operating conditions and that the accuracy of the measurement is adapted to the normal working range of the equipment. Temperature, humidity, vibrations and cleanliness, could affect the accuracy of the calibration process and therefore the measurement of the test gears. In addition to the environmental conditions, other variables of the measuring system must also be taken into account such as axes’ alignments, gear size, test load, rotation speed, data density and the reference artifacts used. Therefore, it is essential to know and control the required environmental specifications both during evaluation and during normal machine use [19,20]. The measurement sensors included in the rolling tester are two linear encoders, two angle encoders and a length gauge. They play a key role in the calibration process, factor that is analysed and presented in this work.

### 2.1. Description of the Worm Gear Tester

A worm gear rolling tester has been developed retrofitting an obsolete gear profile-measuring machine [39]. It integrates single-flank and double-flank tests allowing us to characterize potential error sources in the same kinematic structure, maintaining identical execution conditions in both tests [40]. Although this configuration is outside the usual commercial designs, the operation of the equipment follows the recommendations of the ANSI/AGMA 2111-A98 standard [10] that describes schematically the rolling tests for worm gear transmissions using a master gear.

In particular, the gear rolling tester is able to verify worm gears of up to 600 mm diameter (until accuracy grade 6) using master worms of up to 150 mm diameter and 1000 mm long. This measurement range is wider than usual ranges for this type of gears. Therefore, the errors of the elements involved in the displacements from the initial calibration point to the test point need to be added to the measurement uncertainty of the test itself [41].

**Figure 2:**
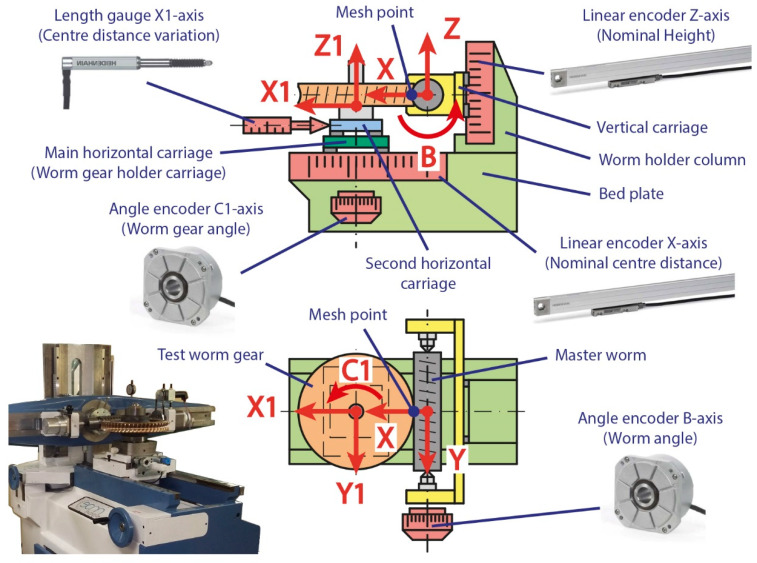
Description of the worm gear rolling tester used.

The tester is composed of three main parts: bed plate, worm holder column and worm gear holder carriage (Figure 2). To perform the single-flank test it is necessary to place the master worm and test worm gear in its nominal test position according to its characteristics and geometry. The test worm gear is moved along the X-axis to the nominal centre distance by the movement of the main horizontal carriage (worm gear holder carriage) sliding on linear guides. The master worm must be moved along the Z-axis to its nominal position in height by sliding the vertical carriage on other similar linear guides. The positions of both carriages are determined by the reading of Heidenhain LF485 linear encoders with an accuracy of ±3 μm. They are incremental linear measuring systems, so position information is obtained by counting individual increments from a reference mark. They operate according to the interferential scanning principle by which a beam of light is diffracted into three partial waves with approximately the same intensity of brightness as it passes through the scanning grid. Most of it is reflected on a graduated ruler passing through the scanning grid again to be deflected and interfered with before reaching the photovoltaic cells at different angles that will convert it into an electrical signal. In the single-flank tests, the rotations of the master worm (B-axis) and test worm gear (C1-axis) are measured by Heidenhain RON 285C angle encoders whose accuracy is ±2.5 arcsec. They are incremental encoders that in this case work according to the imaging scanning principle. A photoelectric element receives a certain amount of light once it has passed through the divisions of a graduated ruler and the scanning grid as they move relative to each other. In the double-flank tests, a second horizontal carriage, superimposed on the main carriage, is released from the nominal position. Its back and forth movement (X1-axis direction) is measured by a Heidenhain ST 1288 length gauge whose accuracy is ±1 μm. It is an incremental linear measurement system that is equipped with a ball-bush guide that works according to the imaging scanning principle.

### 2.2. Error Modelling and Calibration Procedure

The tangential composite deviation parameter in single-flank tests (*Fi’)* is derived from the difference in readings between the master worm angle encoder and the worm gear angle encoder during a complete revolution. The radial composite deviation parameter in double-flank tests (*Fi”)* is extracted from the variation of the centre distance (*a”)* determined by the length gauge. The rest of the decomposed parameters (*Fp’, fi’, Fr”, fi”)* are usually calculated using the Fourier transform [1,2,3]. In both cases, the uncertainty of the measurement process depends on the position where the measurement is made. This is given by the size of the transmission to be checked, which determines the nominal centre distance and the nominal height of the worm gear hobbing plane. It is necessary to add to the measuring instruments’ uncertainty at the calibration point, where two standard cylinders of known diameter are in contact [41], the uncertainty of the meshing point position and orientation due to the displacement to the nominal test position.

In particular, the uncontrolled variability factors such as the distance between centres (Δx), the position in height (Δz) and the perpendicularity between the axes (Δp), condition to a great extent the results of the worm gear rolling tests. For example, errors in the centre distance and height change the contact point between the master gear and worm gear, affecting mainly the tooth-to-tooth rolling parameters. The lack of squareness is equivalent to errors in the hobbing angle of the helix, which changes the functional tooth thickness. However, each influence affects the rolling parameters differently. These are reflected in the estimation of uncertainty by means of sensitivity coefficients that describe how output estimates vary as a function of input estimate values. In this way, it is quantified how each error affects each rolling parameter. Through a proper analysis, it is possible to know the relationship between the accuracy of the measuring instruments, other sources of error and the rolling parameters.

**Figure 3:**
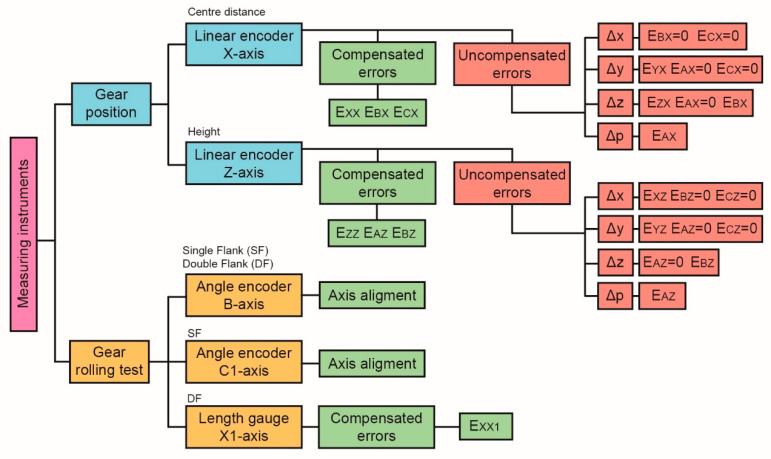
Measuring instruments calibration and evaluation procedure.

In this work, we present a new calibration and evaluation procedure of a single and double-flank rolling worm gear tester, analysing in detail the influence of the measurement sensors used on the accuracy of the equipment. The sequence of the procedure is the following. Firstly, the measuring instruments have been numerically compensated. This process has consisted mainly in comparing their readings with the values obtained by means of laser interferometry and gravity-based method in multiple positions of the axes along five bi-directional approaches [35,36,37]. The main deviations are the result of the cosine error due to the misalignment between the direction of measurement and that of the displacement; of Abbe’s error when carrying out the displaced measurement of the instrument itself; and of the errors generated by the backlash between the elements involved in the movement. The differences obtained are compensated by a correction function in the instrument’s software to minimize the error. Subsequently, a study has been carried out on how the remaining uncompensated errors can affect the theoretical position of the meshing point. This point, where the transmission between worm and worm gear takes place, is located far from the measuring instruments (Figure 2). Therefore, its real position can vary without being perceived by the measuring instruments due to the displacement of the carriages from the calibration point to the possible test positions. For this purpose, both straightness errors and rotation errors have been evaluated. Finally, the residual values of the corrections applied together with the guides geometric errors due to the displacement of the different elements, are included as sources of error in the measurement uncertainty budget [33,34,35]. Figure 3 shows a summary of the procedure followed for each measuring instrument.

Initially, the largest possible gear sizes were considered to be able to estimate the measurement uncertainty of the entire measurement volume. However, it is common for this type of machine to work with more limited measuring ranges. For this reason, a smaller, more reasonable, working volume has also been considered, which allows the estimation of the uncertainty to be adjusted to a more realistic measurement (displacements of 200 mm in the X-axis and 50 mm in the Z-axis for worm gears with a diameter of 400 mm). To this end, a Renishaw XL-80 laser interferometer that provides a linear accuracy of 0.5 ppm and angular accuracy of ±1 μm/m was used for the calibration of the tester. In addition, an electronic level Fowler Wyler Minilevel 54-810-200, with an accuracy of 2%, was also used to measure certain rotation errors. Calibration and evaluation and calibration were carried out under stable conditions of cleanliness and temperature (20 ± 0.5 °C) in the metrological laboratory of a gear manufacturer. These conditions are the same as for the production gears’ testing. Therefore, we consider that the temperature will have a small influence in the measurement uncertainty in comparison with other error sources.

## 3. Results

### 3.1. Linear Encoder (X-axis) Evaluation

#### 3.1.1. Linear Encoder Numerical Compensation

**Figure 4:**
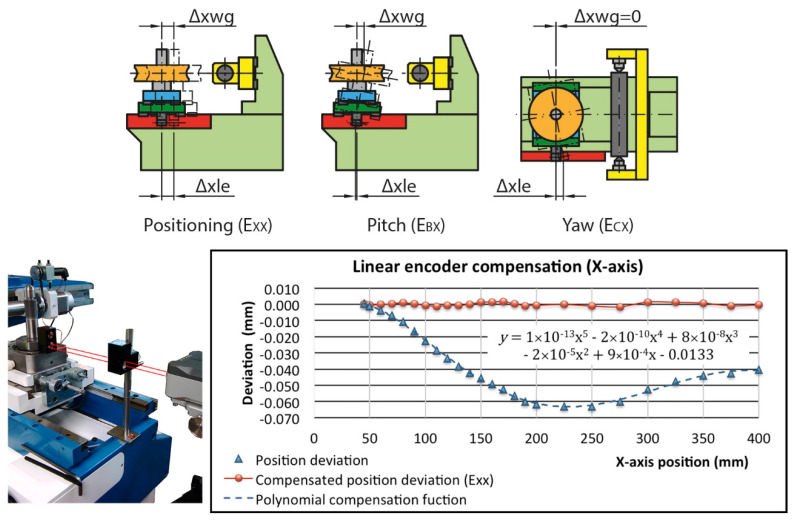
Linear encoder (X-axis) numerical compensation (centre distance position): errors, optics location and results.

The compensation of the main carriage linear encoder (Δx_le_) includes all effects that change the centre distance due to the movement along the X direction (Δx_wg_), i.e., the uncompensated E_XX_ positioning error, part of the pitch E_BX_ and yaw error E_CX_ (Figure 4). For this purpose, we compared the encoder readings with the values provided by the laser interferometer in 10 mm intervals in the tester working area (up to 200 mm) and in 25 mm intervals in the remaining travel. The mobile optics have been placed on the worm gear holder shaft centred with the position of the test worm gear. According to the data obtained, the correction function that best adapts to the behaviour of the worm gear holder carriage, and consequently minimises the positioning error, is a fifth degree polynomial obtained by least squares with a correlation coefficient of 0.99 [37]. With this compensation, a linear (residual) positioning error E_XX_ of 3.2 µm is achieved for the entire travel and 3 µm in the working area (Figure 4). This value will be included as an error source in the uncertainty budget.

#### 3.1.2. Non-Compensated Errors Evaluation

**Figure 5:**
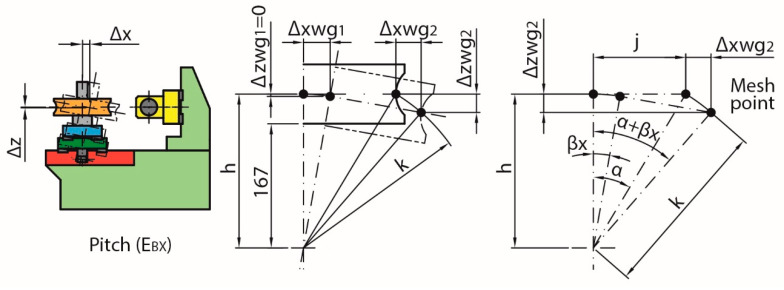
Shift in the worm gear meshing point due to pitch error (E_BX_).

The same positions defined in the numerical compensation along the X-axis have been used to evaluate the uncompensated errors. In addition, it has been considered that the rotation axes of the roll E_AX_ and pitch E_BX_ errors are located in the middle plane of the linear guides used for the main horizontal carriage movement at a distance of 167 mm from the worm gear reference face (Figure 5). The angular errors have been calculated as the sum of the maximum positive and the maximum negative deviation in absolute value. The straightness errors have been calculated according to the maximum deviations with respect to the least squares reference straight line [36].

On the one hand, the pitch error E_BX_ modifies the height of the meshing point, being unnoticed by the linear encoder with the movement of the worm gear holder carriage. The pitch error E_BX_ implies a negligible variation in the height of the worm gear holder carriage shaft (Δz_wg1_). However, this variation is greater at the meshing point because it is further away from the nominal rotation axis (Δz_wg2_). The error will depend on the diameter and height of the worm gear hobbing plane increasing with the size of the worm gear (Figure 5).

Equations (1)–(3) estimate numerically the height variation of the meshing point, as shown graphically in Figure 5.

(1) $$k^{2}=h^{2}+j^{2}$$

(2) $$\alpha =arctan\left(\frac{j}{h}\right)$$

(3) $$\Delta z_{\mathrm{wg}2}=h-k\cdot \cos\left(\alpha +\beta_{x}\right)$$

**Figure 6:**
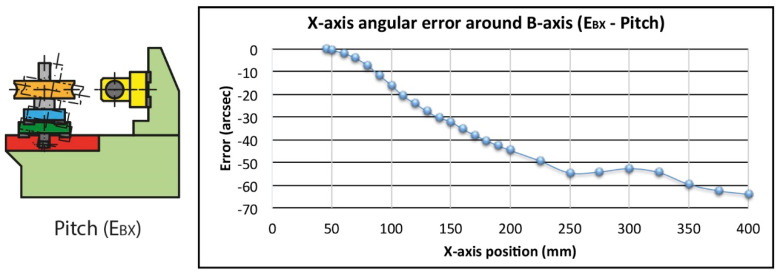
Pitch error (E_BX_).

With:

k, distance from the nominal pitch rotation point to the meshing point;
h, the distance between the linear guides mid-plane and the meshing plane (167 mm + worm gear meshing plane height);
g, worm gear reference radius;
α, nominal meshing point initial angle with respect to the vertical;
β_x_, pitch error E_BX_ (Figure 6);
Δz_wg2_, height variation of the worm gear meshing point due to pitch error E_BX_.

**Figure 7:**
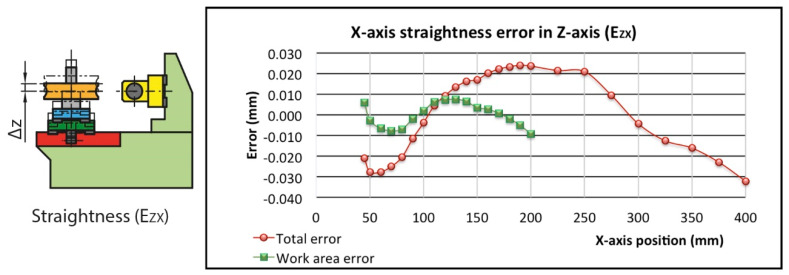
Straightness error (E_ZX_).

The calculations for the largest permissible size worm gear, 600 mm diameter and 100 mm hobbing plane, give a maximum variation of 76 µm in height due to the pitch effect at the meshing point. The value is reduced to 43 µm for a 400 mm diameter worm gear and a mid-plane at 50 mm (Figure 7).

Likewise, if the calculations of the possible variations in the same movement direction are made, Δ_xwg1_ and Δ_xwg2_ (see Figure 5), it can be seen that both values are very similar according to Equations (4) and (5). Thus, their difference is negligible (below 1 µm) so this variation can be considered to be included in the linear encoder compensation and, therefore, does not generate additional uncertainty.

(4) $$\Delta x_{\mathrm{wg}1}=h\cdot \sin\;\left(\beta_{x}\right)$$

(5) $$\Delta x_{\mathrm{wg}2}=k\cdot \sin\left(\alpha +\beta_{x}\right)-j$$

The straightness error E_ZX_ is a direct variation of the distance between the worm axis and the nominal worm gear hobbing plane. In this case, after correcting the misalignment between the laser interferometer and the carriage’s real direction by least squares, the total straightness is 56.2 µm and 16.9 µm in the working area (Figure 7).

**Figure 8:**
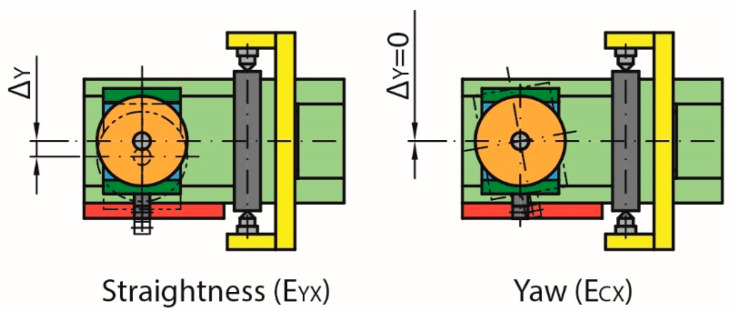
Non-compensated errors (Y-axis) due to movement along the X-axis.

On the other hand, the movement of the worm gear carriage can change the position of the meshing point in the Y direction (Δy) as a result of the straightness error E_YX_ and the yaw error E_CX_ (Figure 8). However, even if this changes the initial meshing point by slightly turning the contact, it does not affect the test results.

**Figure 9:**
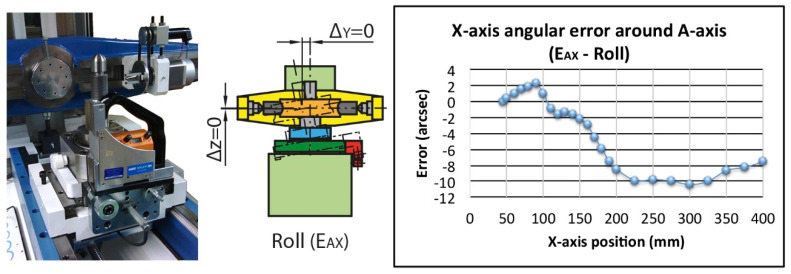
Roll error (E_AX_) and location of electronic level in the measurement.

Finally, the roll error E_AX_ can produce small negligible variations in Y (Δy) and Z (Δz). However, its major influence on the rolling parameters is in the variation of the angle between the Y and Z-axis modifying the squareness of the test without being recorded by the linear encoder. In this case, the roll error E_AX_ is 13 arcsec along the complete travel and 10 arcsec for the working area (Figure 9). An electronic level was used because the rotation around the motion axis (roll) cannot be measured with a laser interferometer [35].

### 3.2. Linear Encoder (Z-axis) Evaluation

#### 3.2.1. Linear Encoder Numerical Compensation

**Figure 10:**
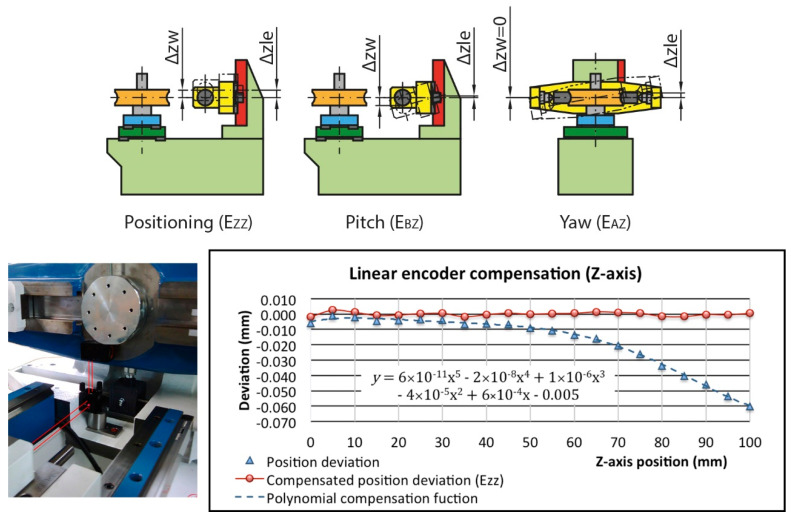
Linear encoder (Z-axis) numerical compensation (height position): errors, optic location and results.

The compensation of the vertical carriage linear encoder (Δz_le_) includes all effects that change the height between the master gear axis and the worm gear hobbing plane due to the movement along the Z direction (Δz_w_), i.e., the uncompensated positioning error E_ZZ_, part of the pitch error E_BZ_ and yaw error E_AZ_ (Figure 10). For this purpose, the encoder readings have been compared with the values provided by the laser interferometer measuring at 5 mm intervals along the entire travel. Due to the limited space on the machine, it was necessary to move the moving optics from the position of the worm, so a correction is applied later. In addition, a rotating mirror optic had to be used to reflect the laser beam to 90°. According to the data obtained, the correction function that best fits the behaviour of the vertical carriage, and consequently minimizes the positioning error, is a fifth degree polynomial obtained by least squares with a correlation coefficient of 0.99. With this compensation, a linear (residual) E_ZZ_ positioning error of 4.3 µm is achieved for the entire travel and 2.3 µm in the working area (Figure 10) which has to be included in the uncertainty budget.

#### 3.2.2. Non-Compensated Errors Evaluation

We used the same positions along the Z-axis as in the numerical compensation to evaluate the errors not compensated by the linear encoder. It has also been considered that the rotation axes of the pitch E_BZ_ and roll E_CZ_ errors are on the mid-plane of the linear guides of the vertical carriage movement. The imperfections in the guides can generate errors, which, although not detected by the measuring instruments and, therefore, not compensated, significantly influence the rolling tests results.

**Figure 11:**
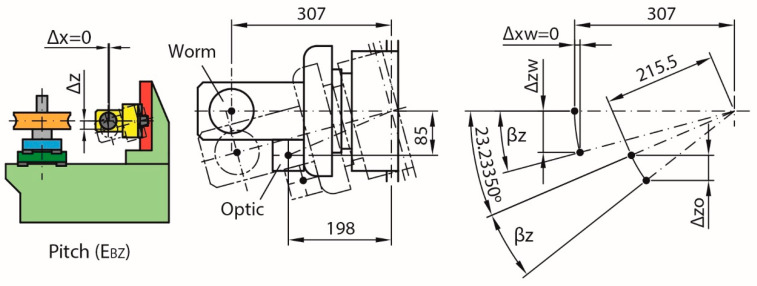
Pitch error (E_BZ_) due to displacement of the master worm carriage.

**Figure 12:**
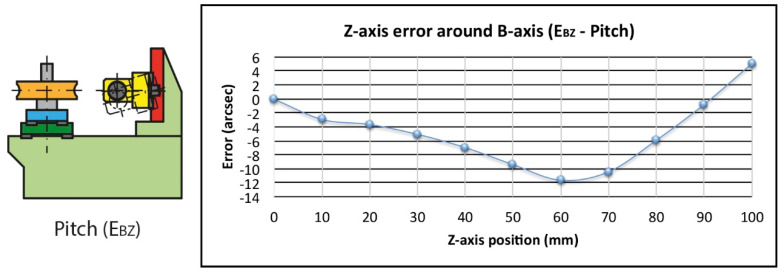
Pitch error (E_BZ_).

The pitch error E_BZ_ was only partially compensated in the calculation of the position error E_ZZ_ due to the location of the optic. The total displacement of the worm in the Z direction (Δz_w_) is greater than that obtained by the laser interferometer reading (Δz_o_) because the distance of the optic’s position from the nominal rotation point is smaller (Figure 11). As the E_BZ_ pitch error (β_z_) is 17 arcsec in the evaluated area (Figure 12), only 16.3 µm out of the potential 25.3 µm height error has been included in the correction. Thus, the difference, 9.0 µm and 2.3 µm considering only the working area, has to be included in the uncertainty calculation (see Equation (6)).

(6) $$\Delta z_{w}-\Delta z_{o}=307\cdot \sin\left(\beta_{z}\right)-215.5\cdot \left[\sin\left(23.23350{}^{\circ}+\beta_{z}\right)-\sin\left(23.23350{}^{\circ}\right)\right]\;=0.0253\;\mathrm{mm}-0.0163\;\mathrm{mm}=0.0090\;\mathrm{mm}$$

**Figure 13:**
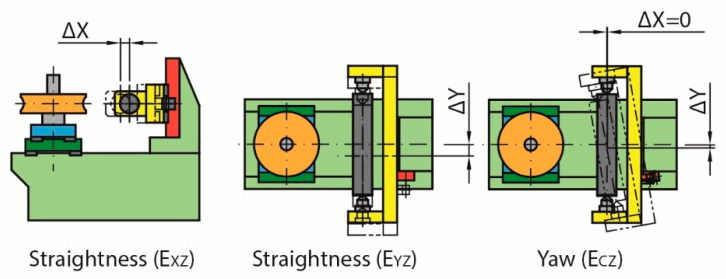
Non-compensated errors (X-axis) due to movement along Z-axis.

On the other hand, the straightness error E_XZ_ is the only one that can change the centre distance without being measured, since the X-direction error due to both pitch error E_BZ_ (Figure 11) and roll error E_CZ_ (Figure 13) during the movement of the master worm carriage can be considered negligible. Even so, we could not measure the straightness E_XZ_ due to the impossibility of properly positioning the optic. In any case, an estimate has been made based on the straightness error E_ZX_ since its design, configuration and components are identical. For this purpose, the highest straightness value equivalent to the evaluated displacement has been considered, which is between the 80 mm and 180 mm in the X axis. For a 100 mm displacement, the maximum straightness error estimated has been 13.1 µm and 4.7 µm for a reduced travel of 50 mm.

The straightness E_YZ_ and roll E_CZ_ errors due to the vertical carriage movement can also change the position of the meshing point in the Y direction (Figure 13). Likewise, there is no effect on the test values because only the contact is slightly rotated and the rolling test results are not affected.

**Figure 14:**
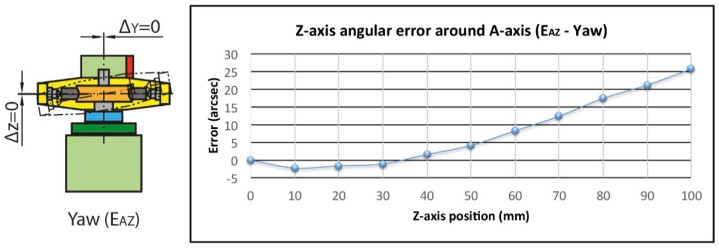
Yaw error (E_AZ_).

Finally, the yaw error E_AZ_ can produce small negligible variations in Y (Δy) and Z (Δz). However, what is remarkable is that it modifies the angle of the worm axis (Y axis) and therefore affects the squareness of the system. In this case, the maximum error measured was 28 arcsec for the total travel and 5.5 arcsec for the working area (Figure 14).

### 3.3. Angle Encoders Evaluation

In the single-flank rolling test, besides the linear measuring instruments which bring the master and worm gears in the nominal position, an angle encoder is required on each rotation axis (B-axis and C1-axis). These compare the angle turned by the worm with that turned by the worm gear, determining the transmission error.

**Figure 15:**
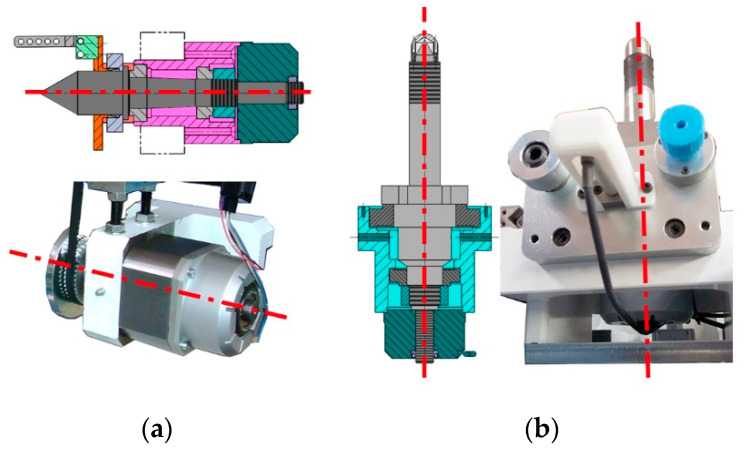
Angular positioning error measurement: (**a**) design detail of the angular encoder alignment with worm rotation axis (B-axis); (**b**) design detail of the angular encoder alignment with worm gear rotation axis (C1-axis).

It is not feasible to calibrate these encoders using an indexing rotation table with an interferometer, a common procedure in machine tool calibration. However, the accuracy of the encoders makes it possible to consider them as reference rotary encoders [36]. This means that they can be used as calibration instruments as long as the machine’s rotary axis is aligned with the rotating part of the encoder and the fixed part of the encoder is connected to the static part of the machine. In this case, both encoders are mounted on a shaft according to the manufacturer’s instructions, so the readings do not have to be compensated for numerically (Figure 15a,b). The uncertainty associated with both the resolution of the encoders and the alignment with the rotation axis can be regarded as included within the uncertainty of the axes rotation.

### 3.4. Length Gauge in the X1-axis Evaluation (Secondary Carriage Position Error)

**Figure 16:**
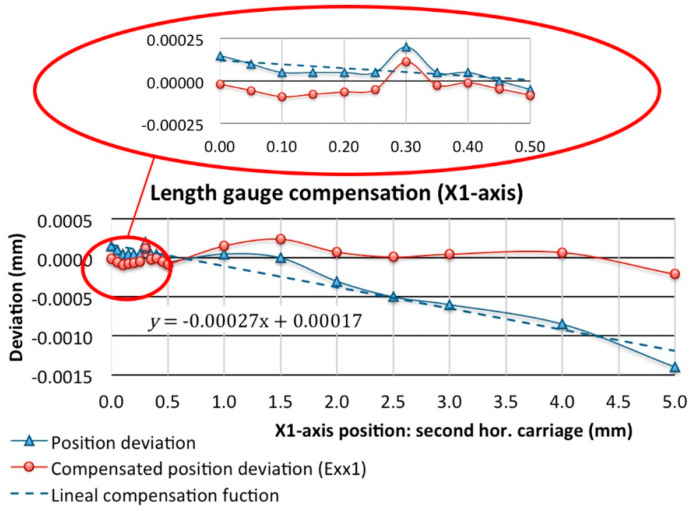
Length gauge compensation (X1-axis).

In the double-flank test, along with the angular encoder for the master worm rotation (B-axis) a length gauge is used to measure the variation of the centre distance along a complete rotation of the worm gear when the secondary horizontal carriage is released. Following the methodology used for linear encoders, the compensation of this measuring instrument has been calculated by intervals of 0.050 mm in the travel range from the blocking position to 1 mm. The rest of the calibration has been undertaken every 0.5 mm up to 3 mm and every 1 mm up to 5 mm of travel. Finally, a linear correction with a correlation coefficient of 0.97 has been applied, obtaining an E_X1X_ position error of 0.4 µm for the whole travel and of 0.2 µm if only 0.3 mm is considered as working area (Figure 16). This residual error is also included as a source of error in the uncertainty budget. As the working range of the secondary carriage is very small, especially compared to that of the main carriage, it is assumed that the remaining errors, both straightness and rotation, can be considered negligible and are included within the measuring instrument error.

## 4. Discussion and Conclusions

Gear rolling measurement techniques have certain advantages over purely geometric methods. For this reason, in recent years new measuring equipment and techniques have been developed on the same principles. However, despite the trend towards international standardization, the ISO standards are limited exclusively to quantifying the gear quality grade and to describing the testing principles in a very general manner, not detailing test conditions or interpretation of results. Nor do they propose how to carry out specific calibration and evaluation of these devices. Moreover, most of the information is related to cylindrical gears, without making special mention of other types of gears. There are no gear rolling standard or accredited national reference laboratories that can guarantee traceability through an unbroken chain of comparisons. The reproducibility of this type of test is, therefore, very limited, despite their high repeatability.

In this context, the uncertainty budget is the most appropriate method to estimate the uncertainty of this measurement process. It allows knowing the individual influence of all sources of error on the rolling parameters. We can obtain the data necessary to estimate the measurement uncertainty by means of experimental characterization and calibration of both the equipment used and the rolling tests. In this way, we could conclude that the uncertainty due to the precision of the instruments is only a part of the total. However, it is only possible to quantify the improvement in reproducibility and in precision when the data are brought into the uncertainty budget. The uncertainty analysis shows that not all errors affect the rolling parameters equally but depend on whether the centre distance, height or angle between the gear axes varies.

In this work, the calibration and evaluation procedure for the measuring instruments used in single and double-flank rolling test equipment for worm gears has been presented. The data obtained are essential to establish the measurement uncertainty of this equipment and, therefore, to determine the maximum gear accuracy grade that could be verified. In particular, the calibration values for the estimation of the uncertainty of the total volume of measurement, but also those of a smaller and usual working volume have been considered. However, the lack of specific regulations together with the perpendicular arrangement of the axes in worm gears make the use of certain standard calibration artifacts unfeasible. Therefore, the decision was to apply techniques commonly used in the calibration and verification of MTs, CMMs and GMIs.

Firstly, the linear measuring instruments (linear encoders and length gauge) have been compensated using laser interferometry. By compensating the linear encoders, it is possible to adjust the positioning readings due mainly to their misalignment and the real displacement of the carriages. Likewise, the compensation of certain angular errors (pitch and yaw) is also partially included. However, this procedure results in residual error values, the positioning errors, which must be included as a source of uncertainty in the budget. The remaining uncompensated errors due to the displacement of the carriages (straightness and angular errors) have been evaluated by calculating how they can affect the meshing point position in the transmission. Some may be considered negligible or having no effect on the rolling parameters but others have a large impact. However, it is only the breakdown of the uncertainty budget that will ultimately show the influence of each error. With respect to the length gauge used in the double-flank test, we performed only the numerical compensation since the rest of the errors have no influence due to the small carriage travel. In addition, the disposition of the angular encoders, aligned with the rotation axis according to the manufacturer’s indications, establishes that calibration is not necessary, it being, therefore, sufficient to consider the measuring instrument’s own error.

The data obtained in this study reveal that rolling test measuring instruments do not register certain variations in the meshing point despite being numerically compensated. As might be expected, they depend both on the gear size and on the errors that occur when moving the components to the nominal test position and, therefore, depend on the machine to be used and on the working area to be verified. However, it is possible to make a valid estimate of values from the calibration errors which, when brought into the uncertainty budget, enables us to know each value’s individual influence on the total uncertainty of the measurement process.

This work intends to show the limitations of measuring sensors in current gear rolling measurement equipment. However, it provides a tool for establishing guidelines for the development of possible new measuring systems, pointing out the most critical points to be considered by means of an uncertainty budget. In addition, it proposes that it would be interesting to establish an ISO standardized calibration and evaluation protocol to complement the current regulatory framework for this type of verification to evaluate the current testers. In this way, the measurement uncertainty could be reduced and the reproducibility could be improved, making it possible to perform faster, more complete and precise verifications with this type of rolling test equipment.
